# GNSS Receiver Identification Using Clock-Derived Metrics †

**DOI:** 10.3390/s17092120

**Published:** 2017-09-15

**Authors:** Daniele Borio, Ciro Gioia, Eduardo Cano Pons, Gianmarco Baldini

**Affiliations:** European Commission, Joint Research Centre (JRC), Directorate for Space, Security and Migration, 21027 Ispra (VA), Italy; ciro.gioia@ec.europa.eu (C.G.); eduardo.cano-pons@ec.europa.eu (E.C.P.); gianmarco.baldini@ec.europa.eu (G.B.)

**Keywords:** fingerprinting, features, Global Navigation Satellite System (GNSS), receiver identification, security

## Abstract

Falsifying Global Navigation Satellite System (GNSS) data with a simulator or with a fake receiver can have a significant economic or safety impact in many transportation applications where Position, Velocity and Time (PVT) are used to enforce a regulation. In this context, the authentication of the source of the PVT data (i.e., the GNSS receiver) is a requirement since data faking can become a serious threat. Receiver fingerprinting techniques represent possible countermeasures to verify the authenticity of a GNSS receiver and of its data. Herein, the potential of clock-derived metrics for GNSS receiver fingerprinting is investigated, and a filter approach is implemented for feature selection. Novel experimental results show that three intrinsic features are sufficient to identify a receiver. Moreover, the adopted technique is time effective as data blocks of about 40 min are sufficient to produce stable features for fingerprinting.

## 1. Introduction

Electronic device fingerprinting is “the process of gathering device information to generate device-specific signatures” [1]. These signatures are then used to identify individual devices or specific device models and introduce an additional layer of security in applications requiring data from a trusted source. The ability to distinguish different device models is usually referred to as inter-model identification, whereas the possibility of identifying individual devices is generally called intra-model identification [1].

Device fingerprinting has several applications including the identification of wireless devices to prevent the forgery of legitimate nodes in a network [2,3,4,5] and the characterization of mobile phones to avoid counterfeiting of electronic components [6,7,8]. Wireless devices can be identified through fingerprinting, and for example, illegitimate Access Point (APs) can be excluded from a wireless network [9,10].

Similar to wireless devices in a network, GNSS receivers are used as data providers in several applications [11,12,13,14]. In the maritime domain, vessels provide their positions to the port authorities through the Automatic Identification System (AIS). Position is obtained using a GNSS receiver that acts as a data provider for the AIS. GNSS data can be easily intercepted, and a genuine GNSS receiver can be easily replaced by a simulator. In this case, receiver fingerprinting can be used to determine the identity of the GNSS receiver used by the AIS and detect illegitimate GNSS data transmissions.

A similar scenario is expected to occur in the road transportation sector, in the Digital Tachograph (DT) context. The DT is an electronic device installed in commercial vehicles, above 3.5 tons in Europe, and is used to record the driving and rest times with the ultimate goal of mitigating the risk of accidents due to a tired driver. EU Regulation 165/2014 [13] foresees the progressive introduction of a new generation of devices, called “smart tachographs”, which will use GNSS and Galileo for improved security and accuracy. In this respect, there may be an economic incentive to tamper with the tachograph and the integrated GNSS receiver. For this reason, EU regulation 165/2014 [13] mandated the usage of the Open Service (OS) Navigation Message Authentication (NMA) that will be provided by the Galileo system. The OS NMA is an authentication system that will enable source verification of the Galileo navigation message. In this case, receiver fingerprinting can be adopted for multi-factor authentication, where the physical properties of the receiver, as identified by its electronic fingerprint, can be used to complement or augment authentication methods such as the Galileo OS NMA [15]. Other examples of multi-factor authentication for electronic devices were presented in [16,17] for WiMax and ZigBee applications.

The data provided by the GNSS receiver integrated in a smartphone can be used for mobile banking services [14]. Thus, there could be an economic interest to falsify the data provided by such receivers. Also in this case, fingerprinting can be used to identify the GNSS data source.

**Figure 1:**
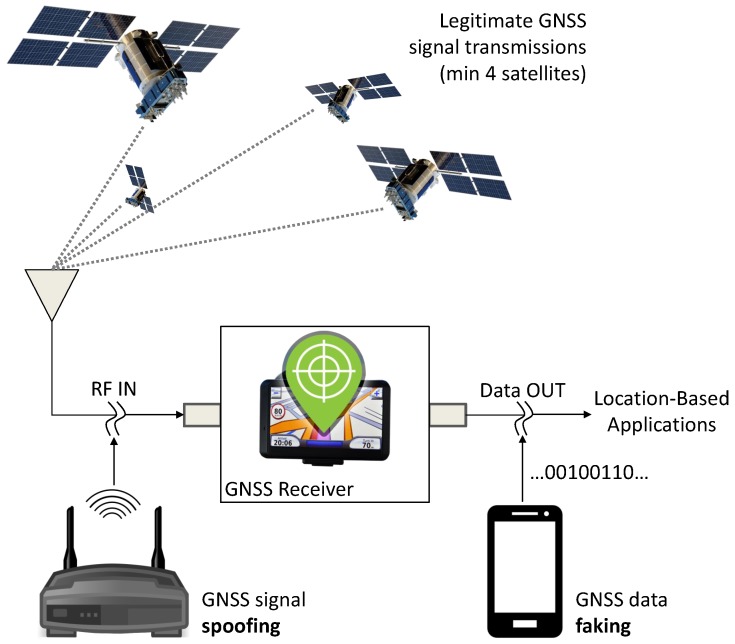
Schematic representation of potential attacks to GNSS receivers leading to forged GNSS data.

A schematic representation of the potential GNSS receiver attacks, which could lead to forged GNSS data, is provided in Figure 1. GNSS signal spoofing [18,19] occurs when legitimate GNSS signal transmissions from GNSS satellites are replaced by synthetic Radio Frequency (RF) signals, generated by a GNSS spoofer, usually located near the receiver. A second vulnerability is GNSS data faking [14], which occurs when the data provided by the receiver itself are intercepted and replaced by false Position, Velocity and Time (PVT) information. In both cases, misleading information is provided to location-based applications, which rely on GNSS receivers as data sources. These types of attacks are different with respect to the vulnerabilities considered in the Wireless Local Area Network (WLAN) domain where devices with full-duplex capabilities are connected to a network [10]. GNSS receiver vulnerabilities and attacks are further reviewed in [19,20]. In this paper, the data at the output of GNSS receivers are considered. More specifically, the paper focuses on the problem of fingerprinting GNSS receivers using the time series provided by the receiver itself at the “Data OUT” node in Figure 1. The work assesses the feasibility of distinguishing different receiver models from experimental data. While the approach developed can be used for improving the security of GNSS data transmission through receiver identification, the problem of detecting attacks involving illegitimate GNSS data is left for future work.

A passive fingerprinting approach [2] is adopted: the methodology developed does not require interrogating the receiver, and its logged data are directly used for device identification. The data provided by the receiver under open-sky conditions are directly used for device identification. Clock-related time series are considered [21]. In particular, GNSS receivers provide the so-called clock bias and clock drift [22]. These quantities are the estimates of the receiver clock offset and drift with respect to the GNSS time scale and characterize the behavior of the receiver’s internal oscillator. Fingerprinting is mainly based on the availability of specific features that are difficult to counterfeit. Clock-derived features have been extensively used to fingerprint physical electronic devices including WiFi cards and other network components. For example, the results presented in [9] demonstrate the possibility of identifying network-connected devices by remotely analyzing the device clock skews. Results from the literature [4,23,24,25] further demonstrate the effectiveness of clock-derived fingerprints for device identification. For this reason, clock-related time series are considered in this paper.

This work extends the preliminary results presented in [21]. In particular, device fingerprinting generally follows three steps [1]: (1) identification of relevant features, (2) extraction and modeling of features and (3) device identification and classification. In this paper, we focus on Step (2) and provide preliminary results with respect to Step (3). In [21], we identified a set of relevant features that were derived from the GNSS clock time series. This aspect is only briefly reviewed in Section 3, whereas the main focus of the paper is the selection and characterization of a subset of features that can be effectively used for fingerprinting. In this respect, we developed a filter method [26,27] to select the most effective features among the candidate metrics discussed in [21]. The method developed is based on the inter- and intra-class distances, and it is better detailed in Section 5. We also investigated the data block duration required for the computation of reliable features. The candidate metrics selected in [21] can be computed from the clock time series considering different data segment durations. Long data segments generally lead to more stable and reliable features. However, it is important to limit the data segment duration for fast device identification. We show that three features are sufficient for device identification. Moreover, these features can be reliably computed using data segments of about 40 min. Although 40 min may seem a prohibitively long time when compared with the time requirements for fingerprinting other electronic devices, such as WiFi cards, it is important to contextualize the application of the proposed techniques and to take into account the technical capabilities of GNSS devices. Telecommunication devices, such as WiFi cards, operate at rates of several Mbps. This means that several millions of samples are collected and processed every second. These high data rates allow fast device fingerprinting. GNSS devices output data at a very limited rate, commonly with a frequency of 1 Hz. Thus, the time required for fingerprinting has to be scaled by the data rate. It is also important to consider the applications where GNSS receivers are employed. In the DT example discussed above, the truck equipped with a GNSS receiver has to be periodically (every two years) brought to a mechanical workshop for inspection, calibration and verification. The analyses at the workshop are significantly longer than 40 min, making the proposed approach practical. In addition to this, it is important to emphasize that GNSS spoofing/data faking attacks require long periods of time. Too fast or abrupt data changes in the receiver position or clock time series make the attack easily detectable. Therefore, the time required by the approach considered in this paper is reasonably lower than the time required to perform the attack.

These aspects were not considered in [21] and are some of the innovative contributions of this paper. Moreover, the experimental analysis has been significantly extended, and new data collections were performed to further support the initial findings discussed in [21]. A new antenna location was selected, and fingerprints were computed using the new experimental data. A good consistency between different data collections is found: this result shows the stability of the metrics selected for fingerprinting with respect to environmental changes.

The analysis conducted and the experimental results obtained show that the receiver model can be effectively determined using clock-derived metrics. While inter-model identification can be easily achieved with the methodology proposed, experimental results show that a different approach is required for intra-model identification.

The remainder of this paper is organized as follows: Section 2 provides a general overview of the system developed for GNSS receiver fingerprinting. Section 3 describes the candidate metrics selected for receiver fingerprinting, and Section 4 introduces the experimental setup adopted for data collection and receiver characterization. The filter method for feature selection is described in Section 5, and experimental results are provided in Section 6. Conclusions are drawn in Section 7.

## 2. System Overview

**Figure 2:**
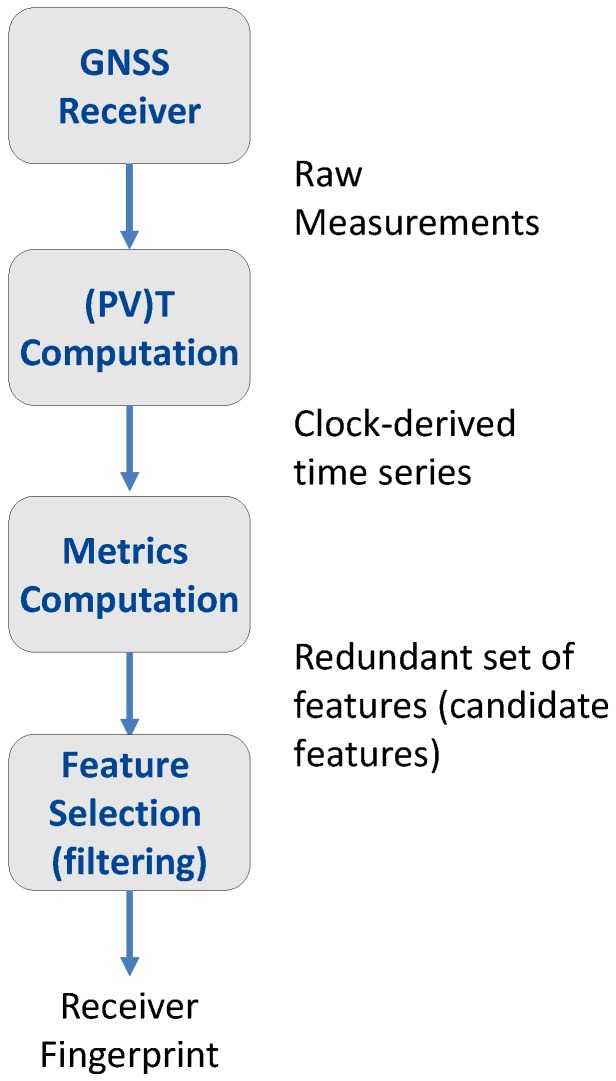
Approach adopted for the determination of the receiver fingerprints.

A comprehensive description of the overall procedure used for the characterization and fingerprinting of the different GNSS receivers is provided in this section. The complete system model is illustrated in Figure 2 and is comprised of the following steps:

- Collection of raw GNSS measurements: The GNSS receivers under test are connected to an antenna in static conditions. We have considered three different locations ranging from the rooftop of a high building under clear-sky conditions to a partially-obstructed environment. The experimental setup, which was used for gathering raw GNSS observables, is better described in Section 4. The data collected include pseudoranges, carrier-phases and Doppler shifts. The clock-related features used for fingerprinting are directly obtained by the observables collected.
- Evaluation of clock-derived time series: The raw observables collected during the previous step are used to estimate the clock biases and drifts of the receivers under tests. Normalized frequency error time series are then derived from the clock drifts and the clock biases.
- Computation of a redundant set of candidate features: Once the normalized frequency error time series are obtained, a large set of clock-related candidate features is computed. These metrics are derived from the computation of the Allan Deviation (ADEV), TIE and the correlation of the normalized frequency errors. A detailed description of the candidate features is provided in Section 3.
- Feature selection: Finally, receiver fingerprints are determined using a filter approach, which consists of choosing the most representative features from the redundant set. The filter procedure adopted in this work is presented in Section 5.

As better detailed in [21], the clock-related metrics considered here can be computed from either the clock bias and clock drift time series. Clock bias time series are computed from pseudoranges [22] and represent the time differences between the local time, generated by the receiver internal clock, and the GNSS time scale, which is usually steered to a Universal Time Coordinated (UTC) realization. Clock drift time series are obtained from Doppler measurements and are estimates of the mismatch between the frequency generated by the receiver local clock and the frequency associated with the GNSS time scale. The clock drift can be considered as the first order derivative of the clock bias, and thus, when clock bias time series are available, it can also be estimated through time differentiation. It is noted that, although of a different nature, Doppler measurements are considered more accurate then pseudoranges [22]. Thus, clock drift computed from Doppler observations are less noisy than their counterpart obtained through the time differentiation of the clock bias.

Most time-related metrics are computed from the so-called “normalized frequency error” [28,29], which is a normalized version of the clock drift. In particular, the estimated clock drift is normalized by the GNSS center frequency, in this case $f_{L_{1}}=1575.42$ MHz. By taking into account the relationship between the clock bias and clock drift, it is possible to express the normalized receiver frequency error as:

(1) $$f_{e}\left[n\right]=\frac{dt_{r}\left[n\right]-dt_{r}\left[n-1\right]}{T_{s}},$$

where $dt_{r}\left[n\right]$ is the clock bias, *n* is the time index and $T_{s}$ is the sampling time of the measurements. The features considered in this paper are computed from $f_{e}\left[n\right]$. 

From the discussion reported above, it emerges that $f_{e}\left[n\right]$ can be estimated either from the clock bias or from the clock drift. Moreover, different approaches can be used for the computation of the clock bias and drift time series. In [21], three approaches were considered for the evaluation of the clock bias and three methods for the evaluation of the clock drift. In particular, the clock bias can be evaluated using a:

- standard approach, where the user position is assumed unknown, and pseudoranges are used to determine the user location and clock bias with a Weighted Least Squares (WLS) approach;
- ’timing’ approach, where the user position is known, and pseudoranges are used to compute the clock bias only; this approach is generally adopted by GNSS timing receivers;
- constrained solution [30], where constrains are used to model the time evolution of the user position.

Similarly, the clock drift can be computed using a:

- standard approach, where Doppler measurements are used to estimate the user velocity and the clock drift; also in this case, a WLS approach is adopted;
- ’timing’ approach, where, similar to the previous case, the user position and velocity are assumed known and constant; thus, Doppler observations are used to estimate only the clock drift;
- constrained solution [30], where constrains are used to model the time evolution of the user position and velocity.

A more detailed description of these approaches can be found in [21].

**Figure 3:**
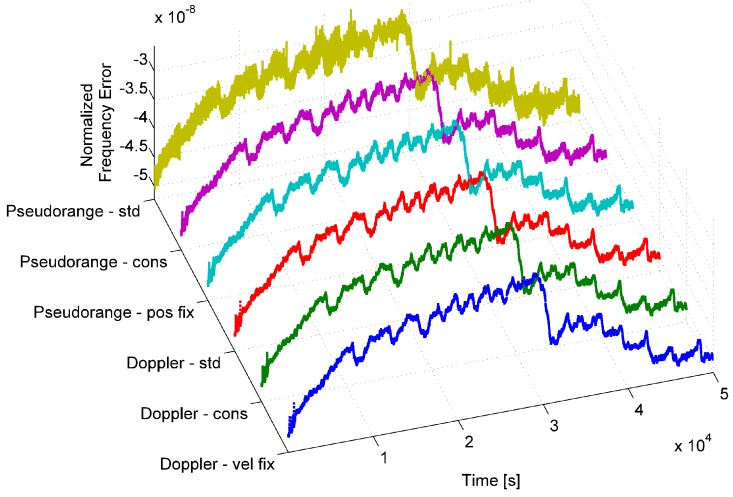
Normalized frequency error time series using Doppler-derived and pseudorange-derived processing strategies.

The normalized frequency error generated using the aforementioned six approaches is plotted in Figure 3 for a u-blox M8T receiver.

From the figure, it emerges that all six time series exhibit the same behavior, and only marginal differences can be noted. The time series obtained by using the estimated clock bias with an unknown position (labeled “pseudorange - std” in Figure 3) is the noisiest, and thus, Doppler-derived time series should be preferred. Furthermore, findings provided in [21] show that the normalized frequency error obtained from Doppler measurements provides features more stable to environmental changes. For this reason, only Doppler-derived time series are considered in the following. Although no significant differences were observed among Doppler-derived time series, we considered the “timing” approach where position and velocity are fixed.

## 3. Candidate Metrics

Several clock-related metrics, widely used in the literature to characterize the behavior of a clock, have been considered in [21] as candidate metrics for fingerprinting. In this section, we provide a short summary of the candidate metrics identified in [21]. These metrics are classified as ADEV-, TIE- and correlation-based metrics. The ADEV is a measure of frequency stability in clocks and oscillators [29]. The ADEV is defined as in [28]:

(2) $$\sigma_{A}\left(\tau \right)=\sqrt{\frac{1}{2(N_{\tau }-1)}\sum_{i=1}^{N_{\tau }-1}{\left({\tilde{f}}_{e,K}\left[i\right]-{\tilde{f}}_{e,K}\left[i-1\right]\right)}^{2}}$$

where τ is the averaging time and is an integer multiple of the of the sampling time:

$$\tau =KT_{s}$$

with *K* an integer value. The time series, ${\tilde{f}}_{e,K}\left[\cdot \right]$, in (2) is a filtered and down-sampled version of the normalized frequency error (1) and is expressed as:

(3) $${\tilde{f}}_{e,K}\left[i\right]=\frac{1}{K}\sum_{n=0}^{K-1}f_{e}\left[iK-n\right].$$

Furthermore, the value $N_{\tau }$ in (2) is the number of frequency error samples available after the filtering and down-sampling processes. The ADEV is a function of the averaging time, τ, and therefore, it cannot be directly used for fingerprinting. However, summary statistics of the ADEV can constitute useful metrics for the fingerprinting process. It was shown in [21] that, for small values of τ (short-term behavior), the ADEV provides more significant information for identifying different receiver clocks. Thus, the ADEV values at τ=1 s and τ=30 s are considered as candidate metrics for fingerprinting. In addition, the slope between these two ADEV values, the minimum value of the ADEV curve and the averaging time corresponding to the minimum ADEV have also been investigated as candidate metrics.

As opposed to the ADEV, which computes the average variations of the normalized frequency errors, TIE-related metrics describe the total phase variation of the frequency errors [28,31]. The TIE is a function dependent on two parameters, the sampling time and the lag *K*, and can be expressed as:

(4) TIE(n,K)=TE[n]−TE[n−K],

where TE[n] is the time error function:

(5) $$TE\left[n\right]=\sum_{i=0}^{n}f_{e}\left[i\right]T_{s}.$$

Two functions, the Root Mean Square TIE (RMS-TIE) and the Maximum TIE (MTIE), can be derived directly from the TIE expression in (4). Also in this case, summary statistics can be selected to model their behavior as a function of *K*, the lag introduced in (4). Similar to the ADEV case, the RMS-TIE and the MTIE functions evaluated at $\tau =KT_{s}=1$ s and $\tau =KT_{s}=30$ s are considered as candidate metrics. In addition, the slopes of RMS-TIE and of the MTIE were selected for evaluation. More detailed information regarding the selection of these metrics can be found in [21]. 

**Figure 4:**
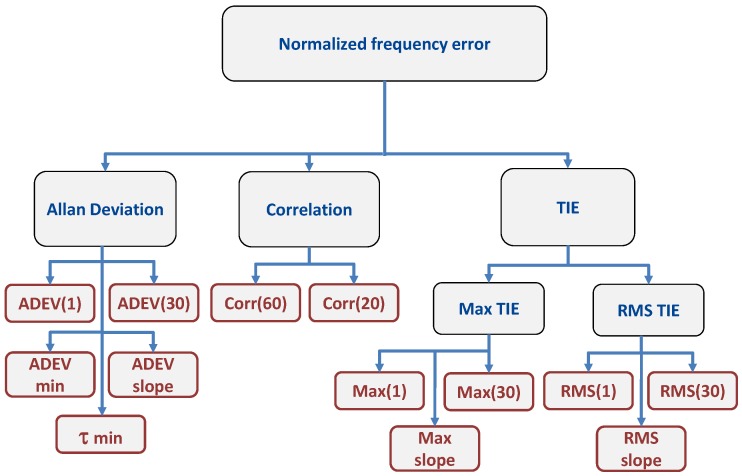
Metrics analyzed for the definition of a fingerprint for GNSS receivers. ADEV, Allan Deviation; TIE, Time Interval Error.

Finally, the correlation function of the normalized frequency error was also analyzed as a source of fingerprinting metrics [23]. The mathematical expression of the normalized correlation function is given by:

(6) $$R\left(\tau \right)=\frac{1}{\sigma_{f}^{2}\left(N-K\right)}\sum_{n=K}^{N-1}\left(f_{e}\left[n\right]-{\bar{f}}_{e}\right)\left(f_{e}\left[n-K\right]-{\bar{f}}_{e}\right)$$

where ${\bar{f}}_{e}$ and $\sigma_{f}^{2}$ are the sample mean and variance of the normalized frequency error [21]. *N* is the total number of samples used for the computation of the correlation function. Two correlation values, at τ=20 s and τ=60 s, were considered as metrics for fingerprinting. A summary of the metrics considered in this paper is provided in Figure 4.

## 4. Experimental Setup

**Figure 5:**
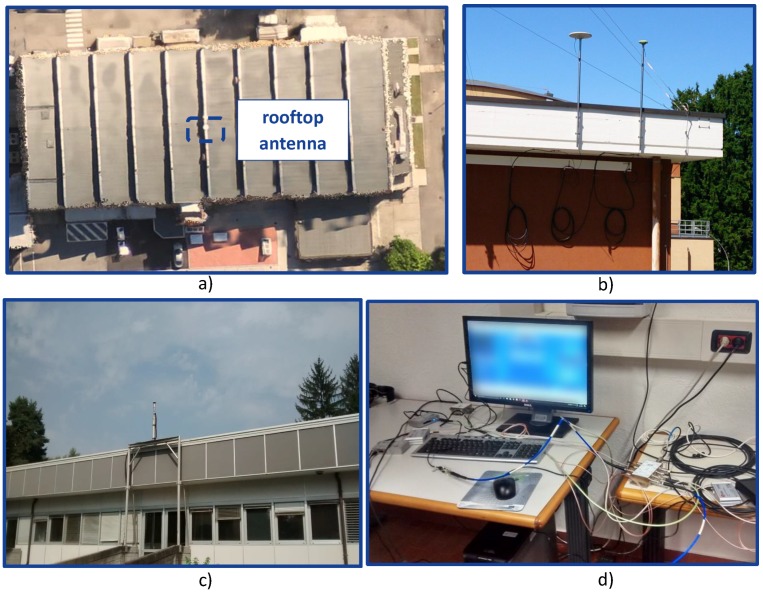
Location of the antennas used for the data collections and laboratory testing setup. (**a**) Clear-sky scenario used for the first data collection. (**b**) Antenna surrounded by buildings and trees used for the second scenario. (**c**) Antenna partially surrounded by trees used for the third scenario. (**d**) Laboratory setup used for collecting and logging GNSS data.

Three data collections were performed in different scenarios in order to evaluate the environmental impact on the metrics used for fingerprinting. The first measurement campaign was conducted using a geodetic antenna located on the rooftop of the European Microwave Signature Laboratory (EMSL) at the JRC premises in Ispra, Italy. This laboratory building is one of the highest on the JRC campus, and no obstacles are present in the vicinity of the antenna. Hence, this first data collection was conducted under clear-sky conditions. The location of the antenna for this scenario is shown in Figure 5a.

The second data collection was carried out using an antenna mounted on the rooftop of an office building within the JRC premises. In this scenario, the office building is surrounded by taller constructions and by high trees, generating a disturbed received signal environment due to multipath and fading effects.

The final measurement campaign was conducted by fixing an antenna on the rooftop of another building on the JRC campus. Furthermore, this scenario is characterized by good signal conditions. Snapshots of the antenna locations for these three measurement campaigns are provided in Figure 5.

A simple laboratory setup was implemented for the three data collections. The GNSS receivers under test were all connected to the same antenna by means of an RF splitter. Dedicated software tools for each receiver model were used for logging and collecting the raw GNSS observables, i.e., pseudoranges and Doppler shifts with a 1 Hz data rate. The GNSS receivers continuously collected data for several days for each data collection. A snapshot of the laboratory testing setup is illustrated in Figure 5d.

Several models of GNSS receivers were used, including professional multi-constellation and mass-market receivers. In order to have the same operational conditions among receivers, only Global Positioning System (GPS) measurements were used since not all of the models offer multi-constellation capabilities. The list of the GNSS receivers that were used for each data collection, including the model’s name and the number of receivers of the same model, is provided in Table 1. A total of five u-blox M8T mass-market receivers were considered: among them, one was updated using the latest firmware provided by the manufacturer. This update was performed in order to investigate the impact of firmware changes on fingerprinting within the same receiver model.

## 5. Data Segmentation and Feature Selection

**Figure 6:**
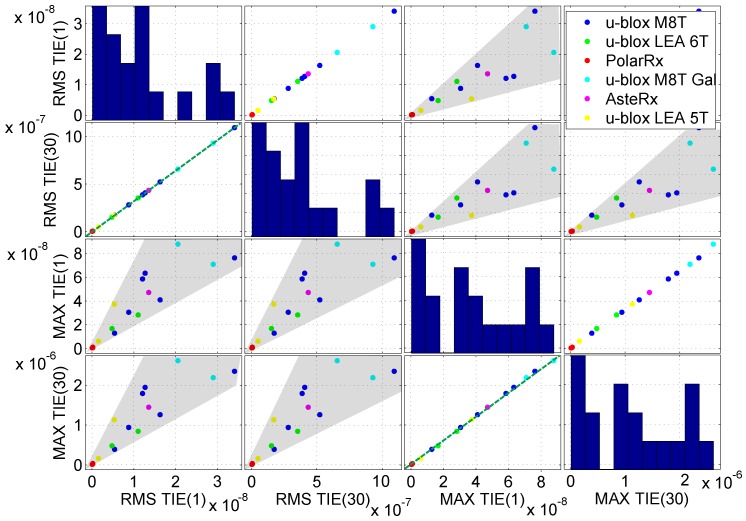
Scatter plots between four candidate metrics considered for receiver fingerprinting.

In Section 3, a set of candidate metrics was presented. These metrics are highly correlated and, in some cases, convey similar information. This fact clearly emerges from Figure 6, which provides a matrix of scatter plots where features are plotted in pairs. In particular, the most correlated candidate features are considered in Figure 6. A clear linear dependence emerges between RMS-TIE(1) and RMS-TIE(30) and between the MTIE(1) and MTIE(30). A dependence between two metrics indicates that only one should be selected for fingerprinting: the second feature does not add any additional information. In this respect, only one RMS-TIE and only one MTIE metric should be considered. Although a strict linear dependence cannot be identified between RMS-TIE and MTIE, Figure 6 clearly shows that an increase of one metric is reflected by an increase of the other.

These considerations have motivated the selection procedure described below. The dependence between metrics was further analyzed in [21], and thus, it is not further considered here.

**Figure 7:**
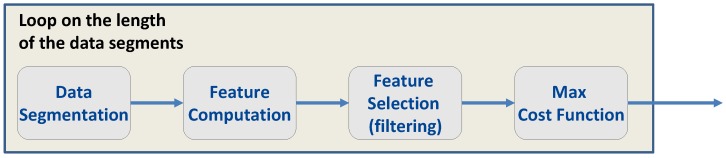
Procedure adopted for data segmentation and for feature selection.

The measurements obtained using the experimental setup described in Section 4 led to long time series where more than 60 h of consecutive data were collected. While it is possible to compute the metrics described in Section 3 using the whole datasets, it is important to determine the minimum duration of the time series such that reliable features can be evaluated. In this way, the required analysis time can be reduced. Thus, the overall procedure depicted in Figure 7 was adopted. The goal of the procedure was two-fold: to establish the duration of the time series for reliable feature evaluation and to select the most effective features for fingerprinting.

In the procedure described in Figure 7, a data duration is at first selected. The clock-derived time series described in Section 2 are then divided into data segments of the duration selected. The data segments are used to compute several realizations of the features described in Section 3, which in turn are used for feature selection.

Several approaches can be adopted for feature selection [26,27]. According to [27], these approaches can be classified as:

- filter methods: Different feature subsets are considered, and for each subset, a score function is evaluated. The score function is used to rank the different subsets. The subset with the highest score is then selected for fingerprinting
- wrapper approaches: These methods require a classifier. Similar to filter approaches, different features subsets are considered, and the classifier is used to score the different subsets. The subset leading to the highest classification performance is selected for fingerprinting
- embedded methods: In this case, feature selection is integrated with the classifier, which determines the best subset of features during the training phase.

Filter methods are usually less computationally demanding than the other approaches and are not tied to a specific classifier. The use of a score function can also be easily adapted to determine the length of the data segments for feature computation. This is the approach used here and depicted in Figure 7: for a fixed data length, the different subsets are considered, and the score function is evaluated. The length of the data segments is determined according to the maximum value achieved by the score function with respect to the different subsets.

**Figure 8:**
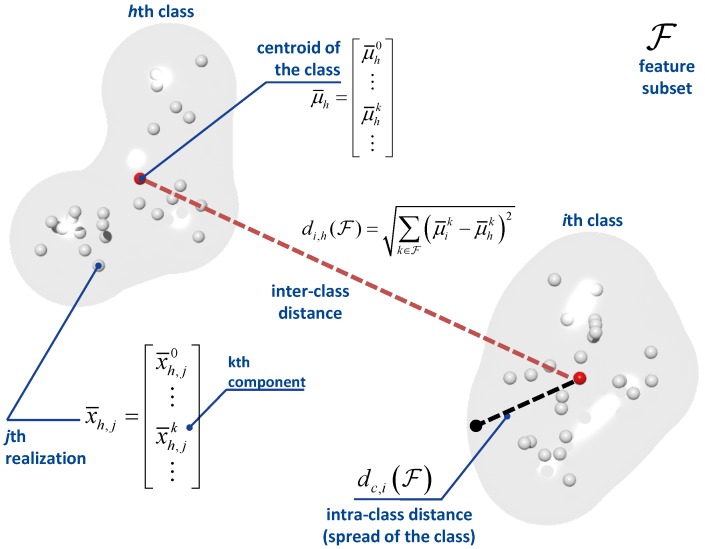
Geometric interpretation of the score function adopted for feature selection.

The score function adopted here is a function of the inter- and intra- class distances. A geometric interpretation of such distances is provided in Figure 8: the inter-class distance quantifies the spread of a class, while the intra-class distance describes separation between two classes.

In order to compute these quantities, it is at first necessary to normalize the feature vector. In particular, let us denote:

(7) $$x_{i,j}=\left[\begin{array}{c} x_{i,j}^{0}\\ x_{i,j}^{1}\\ \vdots \\ x_{i,j}^{k}\\ \vdots \end{array}\right]$$

the *j*-th realization of a feature vector for a receiver belonging to class *i*. The superscript *k* is used to identify the different components of the feature vector. These components are the candidate metrics described in Section 3. Each realization, which is indexed by *j*, is obtained using one of the data segments described above. The components of (7) are heterogeneous in nature, i.e., they assume values in very different ranges, and thus, a normalization is required to make the different features contribute in a similar way to the evaluation of the intra- and inter-class distances. The normalized feature vector:

(8) $${\bar{x}}_{i,j}=\left[\begin{array}{c} {\bar{x}}_{i,j}^{0}\\ {\bar{x}}_{i,j}^{1}\\ \vdots \\ {\bar{x}}_{i,j}^{k}\\ \vdots \end{array}\right]$$

is obtained by applying the following normalization to the individual vector components:

(9) $${\bar{x}}_{i,j}^{k}=\frac{x_{i,j}^{k}-\min_{l,h}\left(x_{l,h}^{k}\right)}{\max_{l,h}\left(x_{l,h}^{k}\right)-\min_{l,h}\left(x_{l,h}^{k}\right)}.$$

In the equations above, the overline notation denotes normalized quantities. The minimum and maximum in (9) are computed considering all feature realizations and all classes. An additional interpretation of (9) will be provided after introducing the notion of intra- and inter-class distances.

For a subset of features, F, the intra-class distance is defined as:

(10) $$d_{c,i}\left(F\right)=\sqrt{\frac{1}{N_{c,i}-1}\sum_{j=0}^{N_{c,i}-1}\sum_{k\in F}{\left({\bar{x}}_{i,j}^{k}-{\bar{\mu }}_{i}^{k}\right)}^{2}}$$

where $N_{c,i}$ is the number of feature realizations for the *i*-th class/receiver type and ${\bar{\mu }}_{i}^{k}$ is the mean feature vector for the *i*-th class. In Figure 8, ${\bar{\mu }}_{i}^{k}$ is the centroid of the class.

The inter-class distance characterizes the separation between two classes, and it is defined as:

(11) $$d_{i,j}\left(F\right)=\sqrt{\sum_{k\in F}{\left({\bar{\mu }}_{i}^{k}-{\bar{\mu }}_{j}^{k}\right)}^{2}}.$$

The subset F is used to select the features to be used in the computation of the distances. Moreover, all of the quantities in (10) and (11) are computed using normalized values (9). In this respect, both (10) and (11) can be interpreted as Mahalanobis distances, where each component is weighted by:

(12) $$w_{k}=\frac{1}{{\left[\max_{l,h}\left(x_{l,h}^{k}\right)-\min_{l,h}\left(x_{l,h}^{k}\right)\right]}^{2}}.$$

The difference, $\max_{l,h}\left(x_{l,h}^{k}\right)-\min_{l,h}\left(x_{l,h}^{k}\right)$, is the range of the *k*-th feature and can be interpreted as a scaled estimate of the feature standard deviation. In this way, (10) can be, for example, rewritten as:

(13) $$d_{c,i}\left(F\right)=\sqrt{\frac{1}{N_{c,i}-1}\sum_{j=0}^{N_{c,i}-1}\sum_{k\in F}w_{i}{\left(x_{i,j}^{k}-\mu_{i}^{k}\right)}^{2}}$$

where non-normalized features are now used. Using (11) and (10), it is finally possible to evaluate the score associated with subset F:

(14) $$G\left(F\right)=\frac{\min_{i\neq j}d_{i,j}\left(F\right)}{\max_{i}d_{c,i}\left(F\right)}.$$

The score for subset F is the ratio between the smallest inter-class distance and the largest intra-class distance. In this way, the maximization of G(F) corresponds to the selection of the feature subset that maximizes the separation between classes.

**Figure 9:**
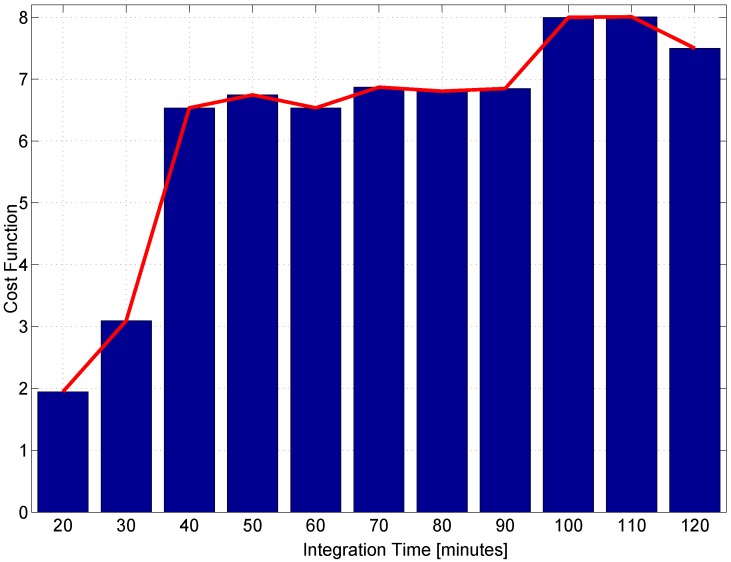
Maximum score function values as a function of the data segment duration.

Score function (14) has been computed considering subsets of Three components. Moreover, data segments with durations ranging from 20 (1200 samples) to 120 min (7200 samples) have been considered. For each duration, the maximum with respect to the feature subsets has been determined. The maximum score values are reported in Figure 9 as a function of the data segment duration. 

From the figure, it emerges that data segments with durations above 40 lead to a score above six. This indicates that features computed with these data durations lead to effective fingerprints for receiver identification. Well-separated classes are obtained, and the minimum inter-class distance is six-times larger than the largest class spread. In the following sections, data segments of duration equal to 60 min (3600 samples) are considered.

## 6. Experimental Results

The data collected according to the experimental setup described in Section 4 have been used to test the approach detailed above. In particular, three aspects have been investigated:

- the stability of the features with respect to environmental changes: in this respect, features obtained from the three antenna locations have been compared;
- the score associated with the different feature subsets for a fixed data segment duration of 1 h (3600 samples);
- the clustering of the different receiver classes when a limited number of features is used for fingerprinting.

**Figure 10:**
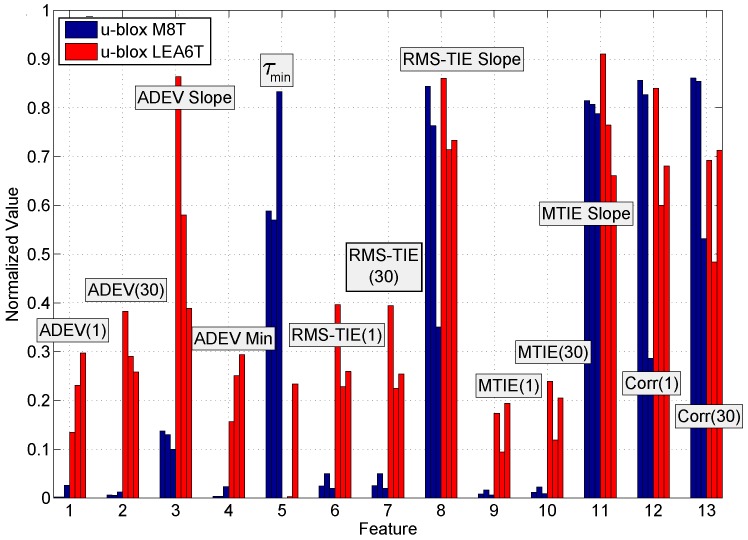
Comparison among features for two different receivers and for the three data collections described in Section 4. For each receiver and for each feature, three bars of the same color are displayed. Each bar corresponds to a different data collection. The feature values have been normalized in the [0,1] range. MTIE, Maximum TIE.

The stability of the candidate features to environmental changes is analyzed in Figure 10 where two receivers are considered as an example. The estimated feature values of two GNSS receiver models, namely u-blox M8T and u-blox LEA6T receiver types, were computed for the three data collections described in Section 4. In order to allow a better graphical representation, features have been normalized according to (9). In this way, all feature components are in the [0,1] range and can thus be better plotted in the same figure. For the two receivers and for each candidate metric, three bar plots are provided corresponding to the three different measurement scenarios. Given a data collection, each bar represents the average value of the normalized feature realization.

Results in Figure 10 show that the two receiver models can be easily distinguished from each other by considering most of the normalized feature values. In particular, the average values for the features ADEV(1), ADEV(30), ADEV slope, $\tau_{min}$, Root Mean Square (RMS)-TIE(1), RMS-TIE(30), MTIE(1) and MTIE(30) show significant differences depending on the selected receiver model.

Figure 10 also shows the consistency of the feature values between different data collections. Most of the features selected have a very consistent behavior in the three cases considered and for each receiver type. This demonstrates the robustness of the features with respect to environmental changes. Similar results were obtained when considering other receivers.

**Figure 11:**
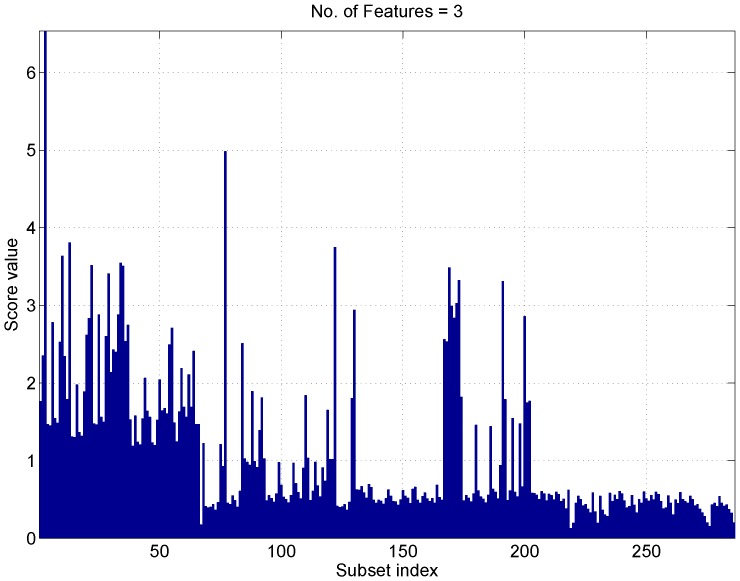
Score results evaluated as a function of the subset index. Subsets of three elements are considered. The analysis is limited to features computed from the clock drift obtained assuming a known location. The data segment duration is of 1 h.

The score associated with the different subsets is analyzed in Figure 11, where subsets of three elements are considered. The subset size was set to three as no significant improvement, in terms of score, was observed when further increasing the dimensionality of the feature vector. When three elements are considered, there are 286 possible subsets from 13 features. These subsets are enumerated by the ’subset index’ in the abscissa of Figure 11. The first 78 subsets include the ADEV(1) and show, overall, scores higher than the other subsets. In this respect, the ADEV(1) is the most discriminating feature from the set considered.

As already mentioned in Section 5, a maximum score higher than six is achieved. This indicates a good separation between the different receiver classes.

**Figure 12:**
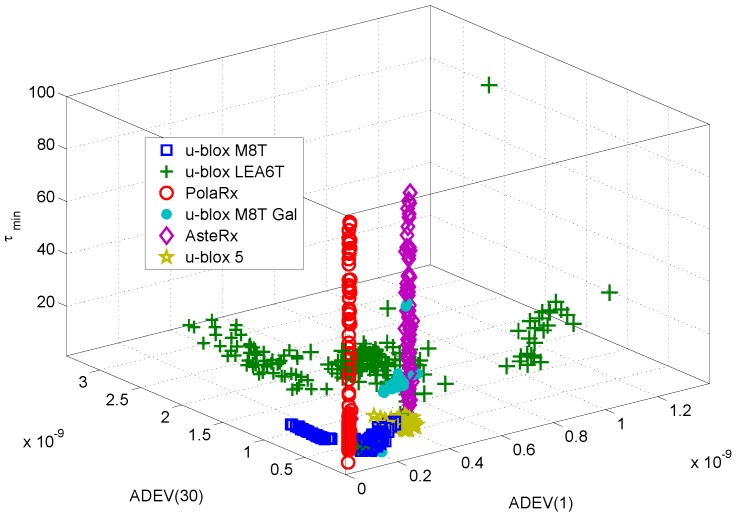
Receiver clustering based on the three features selected from Figure 11. The different receiver types can be easily identified.

Finally, the receiver classes, as represented by their corresponding feature vectors, are shown in Figure 12. The feature vectors depicted in Figure 12 are made of the three components leading to the highest score in Figure 11. The features selected are all based on the ADEV: the ADEV(1), the ADEV(30) and the averaging time, $\tau_{\min}$, corresponding to the minimum ADEV. This result seems to imply that ADEV summary statistics are the most effective for device classification. This result also suggests that other ADEV-derived statistics should be investigated for fingerprinting.

The different receiver classes can be easily recognized from the figure, confirming the fact that three features are sufficient to identify a receiver. It is worth noting that receivers of different models, but from the same manufacturers have similar characteristics. For example, the PolaRx and AsteRx receivers from Septentrio are well localized in the ADEV(1)-ADEV(30) plane. However, a significant variability is observed with respect to the metric $\tau_{\min}$. 

Figure 12 also shows the fact that individual devices are indistinguishable in the feature space considered in Figure 12. This confirms the preliminary result derived in [21] that only inter-model identification is possible with the approach described in this paper.

The separation of the different classes has been further investigated through the implementation of a Gaussian Naive Bayes Classifier (NBC) [32]. It is noted that the detailed analysis of classification performance and the development of specific classification approaches for GNSS receiver identification is outside the scope of this paper. In this respect, the NBC has been implemented only to support the results already presented, confirming the possibility of identifying different receiver types. The NBC has been selected, as it is one of the simplest classification approaches whose implementation depends on a very limited number of parameters. Future developments may investigate the performance of other machine learning classification algorithms.

The data shown in Figure 12 have been used for training and testing the classifier. Several experiments were conducted where, for each round, feature vectors were randomly partitioned into two disjoint sets. About 10% of the fingerprints (i.e., 120 feature vectors) were adopted for training while the remaining data were used to test the capabilities of the NBC. During the training phase, the data were used along with the known class labels to estimate the means and variances of the different transition probabilities used by the NBC. During the test phase, the classifier assigned to each receiver a class label from its feature vector. The three features considered above were provided to the classifier. The predicted class labels were then compared with the corresponding known class labels and the classification performance was analyzed. This type of experiment was repeated several times with different random partitions between training and testing datasets. Results were averaged over the different random partitions.

**Figure 13:**
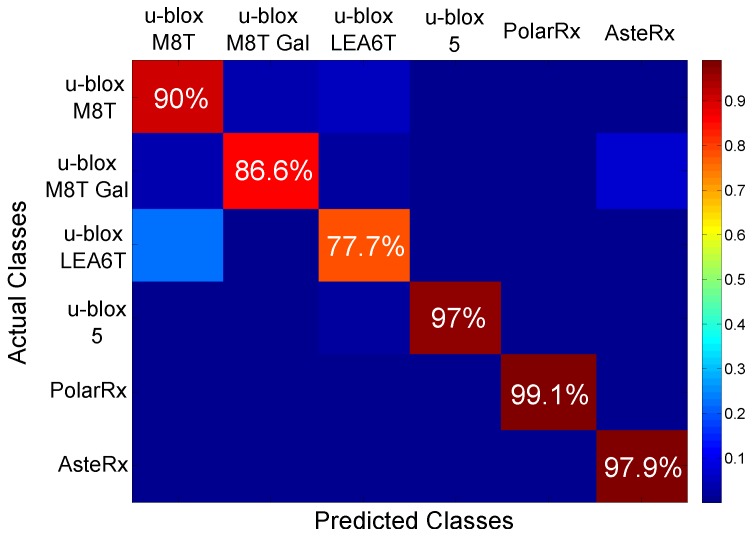
Confusion matrix obtained using a Naive Bayes Classifier (NBC) for receiver classification.

Using this approach, the results shown in Figure 13 were obtained. The figure shows the confusion matrix obtained using the NBC for receiver classification. The values on the diagonal of the matrix are the probabilities of correctly classifying a receiver from its fingerprint. Figure 13 shows that, with the exception of the case of the u-blox LEA6T receiver, correct classification rates are around or above 90%.

These results are in agreement with the findings presented in Figure 12: the classes are well separated, and a classification algorithm can easily identify them. The u-blox LEA6T receiver is the device with the most scattered fingerprints. Moreover, a certain overlapping with the fingerprints of the u-blox M8T devices can be observed in Figure 12. This fact justifies the lower success rate reported in Figure 13. This rate is however close to 80%, and the ambiguity is with respect to a device from the same manufacturer. 

The overall accuracy (i.e., the average of the diagonal elements of the confusion matrix) obtained with the NBC is 91.4%, which is considered a very positive success rate. These results further support the possibility of correctly identifying GNSS receivers from their clock-based fingerprint. 

## 7. Conclusions

This paper describes the results obtained for fingerprinting GNSS devices using clock-based metrics. Features were selected using a data segmentation and filter approach. Feature subsets were considered, and a score was assigned to each subset. The subset with the highest score was selected for fingerprinting. The filter method implemented was also used to select the data segment duration for the computation of the features. From the analysis, it emerges that a fingerprint of three elements is sufficient for identifying the receivers. Moreover, 40 min of data (2400 samples) are sufficient to compute reliable features. Different time series were considered, and it was shown that features should be computed using time series extracted from the receiver clock drift. Clock drift time series are less noisy and provide more reliable results. The experimental analysis conducted considering three different environments shows the stability of the metrics selected and the effectiveness of the approach in determining the receiver model.

## Figures and Tables

**Table 1:** List of the receivers used in the three data collections. The number of receivers used for each model is provided between brackets.

Test 1	Test 2	Test 3
u-blox M8T (4)	u-blox M8T (4)	u-blox M8T (3)
u-blox M8T Galileo (1)	u-blox M8T Galileo (1)	u-blox M8T Galileo (1)
u-blox LEA-6T (1)	u-blox LEA-6T (1)	u-blox LEA-6T (2)
PolarRx (2)	PolarRx (1)	PolarRx (1)
AsteRx (1)	AsteRx (1)	u-blox LEA-5T (1)
	u-blox LEA-5T (1)	

## References

[1] Xu Q., Zheng R., Saad W., Han Z. (2016). Device Fingerprinting in Wireless Networks: Challenges and Opportunities. IEEE Commun. Surv. Tutor..

[2] Shen C., Lu R., Samizade S., He L. Passive fingerprinting for wireless devices: A multi-level decision approach. Proceedings of the IEEE International Conference on Identity, Security and Behavior Analysis (ISBA).

[3] Radhakrishnan S.V., Uluagac A.S., Beyah R. (2015). GTID: A Technique for Physical Device and Device Type Fingerprinting. IEEE Trans. Dependable Secur. Comput..

[4] Azarmehr M., Mehta A., Rashidzadeh R. Wireless device identification using oscillator control voltage as RF fingerprint. Proceedings of the IEEE 30th Canadian Conference on Electrical and Computer Engineering (CCECE).

[5] Padilla P., Padilla J.L., Valenzuela-Valdés J. (2013). Radiofrequency identification of wireless devices based on RF fingerprinting. Electron. Lett..

[6] Baldini G., Dimc F., Kamnik R., Steri G., Giuliani R., Gentile C. (2017). Identification of Mobile Phones Using the Built-In Magnetometers Stimulated by Motion Patterns. Sensors.

[7] Baldini G., Steri G., Dimc F., Giuliani R., Kamnik R. (2016). Experimental Identification of Smartphones Using Fingerprints of Built-In Micro-Electro Mechanical Systems (MEMS). Sensors.

[8] Khanna V.K. (2015). Remote fingerprinting of mobile phones. IEEE Wirel. Commun..

[9] Kohno T., Broido A., Claffy K.C. (2005). Remote physical device fingerprinting. IEEE Trans. Dependable Secur. Comput..

[10] Jana S., Kasera S.K. (2010). On Fast and Accurate Detection of Unauthorized Wireless Access Points Using Clock Skews. IEEE Trans. Mob. Comput..

[11] Papi F., Tarchi D., Vespe M., Oliveri F., Borghese F., Aulicino G., Vollero A. (2015). Radiolocation and tracking of automatic identification system signals for maritime situational awareness. IET Radar Sonar Navig..

[12] Pilosu L., Autolitano A., Brevi D., Scopigno R. Exploring TV white spaces for the mitigation of AIS weaknesses. Proceedings of the IEEE Symposium on Communications and Vehicular Technology in the Benelux (SCVT).

[13] Regulation (EU) No 165/2014 of the European Parliament and of the Council of 4 February 2014 on tachographs in road transport. http://eur-lex.europa.eu/legal-content/EN/TXT/PDF/?uri=CELEX:32014R0165from=EN.

[14] Pujante A. (2014). Paving the Way for New Smartphone Apps. Inside GNSS.

[15] Fernández-Hernández I., Rijmen V., Seco-Granados G., Simon J., Rodríguez I., Calle J.D. (2016). A Navigation Message Authentication Proposal for the Galileo Open Service. Navigation.

[16] Williams M.D., Munns S.A., Temple M.A., Mendenhall M.J. RF-DNA Fingerprinting for Airport WiMax Communications Security. Proceedings of the 2010 Fourth International Conference on Network and System Security.

[17] Dubendorfer C.K., Ramsey B.W., Temple M.A. An RF-DNA verification process for ZigBee networks. Proceedings of the IEEE Military Communications Conference (MILCOM).

[18] Jafarnia-Jahromi A., Broumandan A., Nielsen J., Lachapelle G. (2012). GPS Vulnerability to Spoofing Threats and a Review of Antispoofing Techniques. Int. J. Navig. Obs..

[19] Psiaki M.L., Humphreys T.E. (2016). GNSS Spoofing and Detection. Proc. IEEE.

[20] Ioannides R.T., Pany T., Gibbons G. (2016). Known Vulnerabilities of Global Navigation Satellite Systems, Status, and Potential Mitigation Techniques. Proc. IEEE.

[21] Borio D., Gioia C., Baldini G., Fortuny J. GNSS Receiver Fingerprinting for Security-Enhanced Applications. Proceedings of the 29th International Technical Meeting of The Satellite Division of the Institute of Navigation (ION GNSS+).

[22] Kaplan E.D., Hegarty C. (2005). Understanding GPS: Principles and Applications.

[23] Polak A.C., Goeckel D.L. (2015). Wireless Device Identification Based on RF Oscillator Imperfections. IEEE Trans. Inf. Forensics Secur..

[24] Lanze F., Panchenko A., Braatz B., Zinnen A. Clock skew based remote device fingerprinting demystified. Proceedings of the IEEE Global Communications Conference (GLOBECOM).

[25] Sharma S., Hussain A., Saran H. (2012). Experience with Heterogenous Clock-skew Based Device Fingerprinting. Proceedings of the 2012 Workshop on Learning from Authoritative Security Experiment Results.

[26] Guyon I., Elisseeff A. (2003). An Introduction to Variable and Feature Selection. J. Mach. Learn. Res..

[27] Chandrashekar G., Sahin F. (2014). A survey on feature selection methods. Comput. Electr. Eng..

[28] Bregni S. (2002). Synchronization of Digital Telecommunications Networks.

[29] Allan D.W. (1966). Statistics of atomic frequency standards. Proc. IEEE.

[30] Xu G. (2003). GPS: Theory, Algorithms and Applications.

[31] Bregni S. (1996). Measurement of Maximum Time Interval Error for Telecommunications Clock Stability Characterization. IEEE Trans. Instrum. Meas..

[32] Murty M.N., Devi V.S. (2011). Pattern Recognition: An Algorithmic Approach.

